# Studying the Co-presentation of Focal Segmental Glomerulosclerosis and IgA Nephropathy in a Young Female With No Significant Risk Factors

**DOI:** 10.7759/cureus.37212

**Published:** 2023-04-06

**Authors:** Omar Elsemary, Iqbal S Grewal, Saifullah Nasim, Sohail Nasim

**Affiliations:** 1 College of Medicine, California Northstate University College of Medicine, Elk Grove, USA; 2 Internal Medicine, California Northstate University College of Medicine, Elk Grove, USA; 3 Nephrology, West Hills Hospital, West Hills, USA

**Keywords:** disease progression, internal medicine, kidney disease, rare disease, nephrology, medicine

## Abstract

Focal segmental glomerulosclerosis (FSGS) and IgA nephropathy are among the most common glomerular disorders. FSGS is characterized by focal scarring affecting less than 50% of glomeruli while IgA nephropathy is characterized by the deposition of IgA in the mesangium of glomeruli. The presence of both of these diseases in a single patient is uncommon, but the presence of both in a young individual with no predisposing factors is exceedingly rare. As such, our case report outlines the unusual presentation of both of these disorders in a young Hispanic female with no known risk factors.

## Introduction

Focal segmental glomerulosclerosis (FSGS) is a condition that occurs once scar tissue develops in the kidney, impeding its ability to filter waste from the blood. In extreme cases, it may lead to kidney failure, for which the only treatment options are dialysis or kidney transplant. FSGS is the most common primary glomerular process contributing to end-stage renal disease [1]. It is far less common for young individuals to experience FSGS with IgA nephropathy and acute tubular necrosis. Our case report is about a young Hispanic female who developed FSGS concomitant with IgA nephropathy and acute tubular necrosis with no evidence of significant predisposing factors.

## Case presentation

A 22-year-old Hispanic female with no known medical problems presented to the Emergency Department (ED) with a three-day history of worsening generalized crampy abdominal pain. She had been experiencing similar, albeit less severe, pain intermittently over the past year and was taking ibuprofen 600 mg once a day two to three times a week since the onset of symptoms. In addition, she reported nausea, vomiting, and diarrhea since the onset of abdominal pain. Her history was also remarkable for increased urinary frequency for the last two years without accompanying dysuria, urgency, or hematuria.

The patient had presented to the ED a year earlier with a complaint of abdominal pain. Laboratory evaluation was remarkable for blood urea nitrogen (BUN) of 25 mg/dL (normal = 7-18 mg/dL) and serum creatinine level of 2.88 mg/dL (normal = 0.55-1.30 mg/dL).

The patient denied a family history of hypertension, diabetes mellitus, kidney disease, or autoimmune diseases. She also denied tobacco use and stated that she occasionally consumed alcohol. Physical examination revealed an elevated blood pressure (146/85 mmHg), a body mass index of 26.1 kg/m^2^, lungs clear to auscultation, and no pitting edema. The patient’s hepatitis panel and HIV test were negative. Her laboratory data are shown in Tables 1-3.

**Table 1:** Urinalysis results.

Test	Result	Normal
Urine color	Straw	Yellow
Urine clarity	Cloudy	Clear
Urine pH	5.0	5.0–7.0
Urine protein	≥500 mg/dL	Negative
Urine blood	+1	Negative
Urine red blood cell	6–10/HPF	0–2/HPF

**Table 2:** Complete blood count results.

Test	Result	Normal values
White blood cell	10,040/mm^3^	4,000–10,500/mm^3^
Red blood cell	3.38 million/mm^3^	4.2–5.4 million/mm^3^
Hemoglobin	9.6 g/dL	12.0–16.0 g/dL
Hematocrit	30.3%	36–47%
Mean corpuscular volume	89.6 fL	80–98 fL

**Table 3:** Serum chemistry results.

Test	Result	Normal values
Sodium	139 mEq/L	136–145 mEq/L
Potassium	3.7 mEq/L	3.5–5.1 mEq/L
Chloride	110 mEq/L	98–107 mEq/L
Blood urea nitrogen	42 mg/dL	7–18 mg/dL
Creatinine	5.18 mg/dL	0.55–1.30 mg/dL
Estimated glomerular filtration rate	11 mL/minute	≥60 mL/minute
Calcium	8.2 mg/dL	8.5–10.1 mg/dL

Laboratory results were most remarkable for microscopic hematuria, 4+ proteinuria, normocytic anemia, elevated BUN and creatinine levels, and a markedly low estimated glomerular filtration rate (GFR).

**Figure 1:**
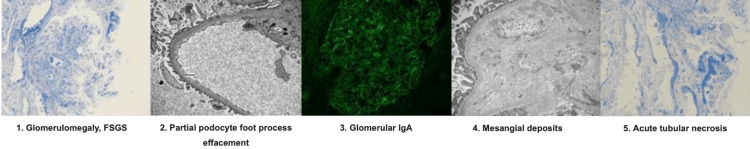
Biopsy and imaging results. 1: Light microscopy findings of focal segmental glomerulosclerosis. 2: Electron microscopy findings of partial podocyte foot process effacement. 3: Immunofluorescence microscopy findings of IgA deposition in the glomeruli. 4: Small paramesangial (immune complex-type) electron-dense deposits. 5: Light microscopy findings of non-atrophic tubules displaying acute tubular necrosis.

Ultrasound revealed that both kidneys were small with increased cortical echogenicity and poor corticomedullary differentiation. Mild fullness of the collecting system was noted which was more pronounced on the left. Nuclear medicine renal flow/function test showed increased excretion time with decreased right renal function relative to the left. A renal biopsy was performed. On light microscopy, four glomeruli were seen and all were globally sclerosed. Some tubular segments displaced acute tubular necrosis and compensatory hypertrophy. There was moderate tubulointerstitial scarring associated with mild interstitial mononuclear infiltrates. Immunofluorescence microscopy was performed with fluoresceinated antisera to human IgG, IgM, IgA, C1q, C3, kappa, lambda, and albumin. There were six glomeruli, of which two were globally sclerosed. In the remaining glomeruli, there was focal glomerulomegaly and fine-to-coarse mesangial staining for IgA (3+), IgM (trace), C3 (1+), kappa (2+), and lambda (1 to 2+). Electron microscopy revealed four glomeruli, of which one was globally sclerosed and two were segmentally sclerosed. Results are displayed in Figure 1.

Following the initial workup, FSGS and IgA nephropathy were the leading diagnoses and appropriate treatment was begun. The patient was started on prednisone 60 mg QD, cinacalcet 30 mg QD, calcitriol 0.25 µg QD, cholecalciferol 10,000 U QD, and iron 324 mg BID and was discharged from the hospital with instructions for follow up with outpatient primary care physician and outpatient nephrology in eight weeks to monitor status and make treatment changes based on response to the current regimen. Unfortunately, the patient was lost to follow-up and did not return to the outpatient nephrology appointment.

## Discussion

The prevalence of end-stage renal disease has been dramatically increasing in the United States, having more than tripled since 1990. The Centers for Disease Control and Prevention estimates that about 800,000 people are being treated for end-stage renal disease, making it safe to assume that the prevalence is, in fact, even higher than this [2]. The exact definition of end-stage renal disease remains unclear; however, it has recently been defined as a terminal illness with a GFR of less than 15 mL/minute [3]. Many disease processes lead to end-stage renal disease, one of which is glomerular diseases such as FSGS. In the United States, FSGS is the most common cause of end-stage renal disease caused by glomerular diseases [4]. To diagnose FSGS, laboratory values, such as proteinuria, are used in conjunction with histopathology to visualize the glomeruli. The presentation of FSGS can be widely variable and may include the manifestation of nephrotic syndrome, hypertension, and hematuria [1]. Upon obtaining a kidney biopsy, patients with FSGS will have scarring across parts of the glomerulus, affecting only some of the glomeruli that are sampled [1]. Although the prevalence of FSGS has been rising steadily in recent years as a whole, men are more commonly affected than women. Additionally, there is a significantly higher proportion of African Americans and Hispanics affected by this condition in comparison to Caucasians [5].

IgA nephropathy is the most common form of glomerulonephritis worldwide [3]. There is a wide geographical variation in disease prevalence. The highest incidence of the disease is present in East and Pacific Asian countries, but data must be evaluated with caution as practices with biopsy, dialysis, and overall heterogeneity of data sources around the world. The presentation may occur at any age but is most common in the second or third decade of life [6]. Patients present with either macroscopic hematuria and/or an upper respiratory tract infection. It is much more uncommon to present with acute kidney injury as a result of gross hematuria causing a tubular obstruction. Nephrotic-range proteinuria can be seen in more severe cases. Clinical findings may include elevated serum creatinine, reduced GFR, hypertension, and proteinuria in the nephritic range. Definitive diagnosis is established from a kidney biopsy with immunofluorescence. The report shows globular deposits of IgA, accompanied by C3 and IgG, in the mesangium. The staging of the disease can be determined from the Oxford classification, which is an indicator of severity and outcome [7]. Light microscopy shows a segmental area of endocapillary proliferation within the glomerular tuft.

FSGS and IgA nephropathies co-existing in one patient is not a common occurrence and there has been little reported literature explaining its pathophysiology or its treatment. The co-existence of these conditions indicates that one will experience symptoms, clinical findings, and biopsy findings that overlap with both FSGS and IgA nephropathy.

IgA nephropathy co-existing with rapidly progressive glomerulonephritis has had observational studies that look at treatment plans. The studies utilized intravenous (IV) methylprednisolone, followed by oral prednisone, IV cyclophosphamide, or plasmapheresis [8]. Medications to treat the symptoms should be given to alleviate any other problems that could arise. This can be medications such as angiotensin-converting enzyme inhibitors or angiotensin receptor blockers for hypertension. For any other uremic findings, patients should be given the respective medication to help the ailment.

## Conclusions

Our case focuses on a 22-year-old Hispanic female to call attention to the increasing prevalence of the co-presentation of FSGS and IgA nephropathy in the United States. This presentation is relatively rare, especially considering our patient’s age and gender, and is associated with poor outcomes, further highlighting its importance. Furthermore, the administration of prednisone has proven to be effective in other complex presentations of end-stage renal disease, making it a possible treatment method to be explored in patients with co-existing FSGS and IgA nephropathy.
